# Advances in Wood Processing, Flame-Retardant Functionalization, and Multifunctional Applications

**DOI:** 10.3390/polym17192677

**Published:** 2025-10-03

**Authors:** Yatong Fang, Kexuan Chen, Lulu Xu, Yan Zhang, Yi Xiao, Yao Yuan, Wei Wang

**Affiliations:** 1 Fujian Provincial Key Laboratory of Functional Materials and Applications, School of Materials Science and Engineering, Xiamen University of Technology, Xiamen 361024, China; cube-leaf@163.com (Y.F.); chenkexuano@163.com (K.C.); 2020000004@xmut.edu.cn (Y.X.); 2 School of Chemical Engineering, University of New South Wales, Sydney, NSW 2052, Australia; lulu.xu1@unsw.edu.au; 3 Zhejiang Provincial Engineering Research Center for Green and Low-Carbon Dyeing & Finishing, Ministry of Education, Zhejiang Sci-Tech University, Hangzhou 310018, China; zy52360@zstu.edu.cn; 4 School of Engineering, RMIT University, Melbourne, VIC 3001, Australia

**Keywords:** wood materials, modification, environmentally friendly, flame retardancy, flame retardant mechanism

## Abstract

Wood is a renewable, carbon-sequestering, and structurally versatile material that has supported human civilization for millennia and continues to play a central role in advancing sustainable development. Although its low density, high specific strength, and esthetic appeal make it highly attractive, its intrinsic flammability presents significant challenges for safety-critical uses. This review offers a comprehensive analysis that uniquely integrates three key domains, covering advanced processing technologies, flame-retardant functionalization strategies, and multifunctional applications. Clear connections are drawn between processing approaches such as delignification, densification, and nanocellulose extraction and their substantial influence on improving flame-retardant performance. The review systematically explores how these engineered wood substrates enable more effective fire-resistant systems, including eco-friendly impregnation methods, surface engineering techniques, and bio-based hybrid systems. It further illustrates how combining processing and functionalization strategies allows for multifunctional applications in architecture, transportation, electronics, and energy devices where safety, durability, and sustainability are essential. Future research directions are identified with a focus on creating scalable, cost-effective, and environmentally compatible wood-based materials, positioning engineered wood as a next-generation high-performance material that successfully balances structural functionality, fire safety, and multifunctionality.

## 1. Introduction

Wood is among the oldest structural materials known to humankind and remains one of the most versatile [1,2,3,4,5,6]. It is widely appreciated for its renewability, low embodied energy, favorable strength-to-weight ratio, and inherent esthetic qualities. With the growing emphasis on sustainability and carbon neutrality, wood is receiving renewed attention as an alternative to energy-intensive materials like steel, aluminum, and concrete [7,8,9,10,11,12]. Unlike petroleum-based resources, wood is carbon-neutral because the carbon fixed during photosynthesis is balanced by its release during natural degradation or combustion. This cyclical carbon balance positions wood as a central material in strategies for reducing greenhouse gas emissions. Despite these advantages, the broader use of wood in advanced engineering applications is restricted by several inherent limitations. Its organic composition makes it highly susceptible to fire, since cellulose and hemicellulose thermally decompose into flammable volatiles [13,14,15,16,17]. The hygroscopic nature of wood leads to swelling and shrinkage under variable humidity, undermining dimensional stability. Furthermore, its susceptibility to fungal, insect, and microbial attack reduces long-term durability, and its mechanical performance, while excellent relative to density, remains inferior to that of many modern composites. These challenges underscore the need for targeted processing and functionalization strategies to extend the service life of wood, enhance its reliability, and unlock its potential as a sustainable replacement for conventional petroleum-derived materials [18,19].

Overcoming these limitations requires a shift from conventional preservation and surface treatments toward advanced functionalization strategies. Recent progress in wood science, driven by advances in chemistry, nanotechnology, and materials engineering, has created unprecedented opportunities to manipulate wood’s structure at the micro- and nano-scale [20,21,22,23]. Processes such as delignification, densification, and surface engineering enhance mechanical strength and stability while providing versatile scaffolds for introducing new functionalities. At the same time, the development of flame-retardant wood has become a central focus, with strategies evolving from passive coatings to integrated molecular and nanoscale modifications that improve fire resistance and maintain environmental compatibility [24,25,26,27,28,29,30,31,32].

Over the past few decades, wood processing and functionalization have advanced at an unprecedented pace, fueled by innovations in chemistry, nanotechnology, and materials science. A growing suite of techniques, including delignification [33,34,35], densification [36,37,38], chemical modification [39], and surface engineering [40,41], has made it possible to reconfigure the hierarchical structure of wood, fine-tune its micro- and nanoscale architecture, and tailor its physicochemical properties with remarkable precision. These approaches not only strengthen mechanical performance and improve resistance to moisture and biological attack but also create a versatile platform for imparting functions that extend far beyond those of natural wood. Among these developments, flame-retardant functionalization has emerged as particularly transformative, addressing the critical challenge of flammability that has long limited wood’s wider adoption in advanced applications [42]. By employing strategies such as molecularly engineered flame-retardant moieties, the integration of inorganic and organic components through hybrid systems, and the use of sustainable bio-based formulations, researchers have succeeded in producing wood materials that combine enhanced fire safety with environmental compatibility, thereby paving the way for safer, greener, and more multifunctional uses of this ancient yet continually evolving material [43,44].

Beyond safety, the development of flame-retardant wood has advanced toward multifunctional systems that overcome the inherent limitations of natural wood. Through structural design, chemical treatment, and the integration of flame-retardant fillers or nanomaterials, researchers have achieved significant enhancements in thermal stability, mechanical robustness, smoke suppression, and durability, while simultaneously introducing additional functionalities such as ultraviolet resistance [45,46], antimicrobial performance [47], and improved dimensional stability [48]. These innovations expand the practical applications of wood while positioning it as a sustainable and high-value material platform for addressing challenges in fire safety and environmental protection. By coupling sustainability with advanced performance, flame-retardant wood is emerging as a promising material for green construction, safe transportation, and energy-efficient infrastructure, thereby redefining its role in modern materials science and contributing to next-generation sustainable technologies.

Although a substantial body of research exists in this field, existing reviews generally address wood processing, flame retardancy, and functionalization as separate topics. This highlights the need for a comprehensive overview that systematically connects these interrelated areas. The present review fulfills this need by offering a unified, mechanism-focused comparison of bulk and surface flame-retardant approaches across different processing pathways. Our approach distinguishes itself from previous reviews in both scope and methodology, as it synthesizes technical advancements while providing a broader perspective on their implications. This integrated viewpoint offers considerable value. For researchers, it delivers a structured understanding that can guide the development of more effective and holistic materials. For industrial practitioners, it emphasizes crucial considerations such as long-term durability, scalability, and alignment with building codes, which are essential for the successful real-world application of advanced wood materials.

**Figure 1:**
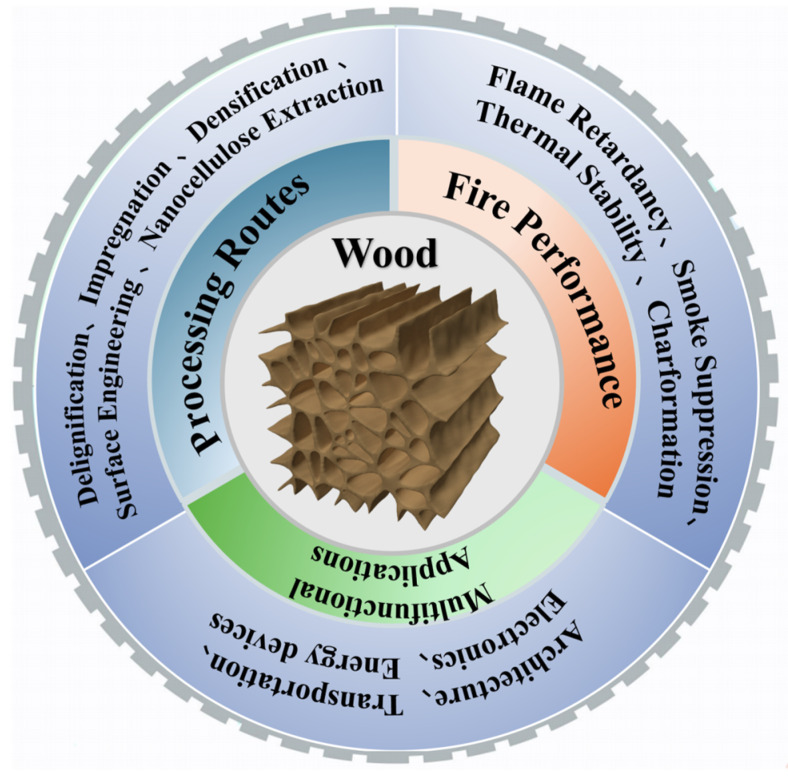
Overview of wood processing strategies, flame-retardant performance, and multifunctional applications.

Through the convergence of structural engineering, flame-retardant strategies, and multifunctional modifications, researchers are reshaping wood from a traditional natural resource into a new class of sustainable, high-performance, and intelligent material system capable of meeting the complex demands of modern technology and society. This review provides a comprehensive overview of these advances, beginning with a discussion of emerging wood processing technologies that allow precise control of micro- and nanoscale structure to enhance strength, stability, and durability. It then examines state-of-the-art flame-retardant functionalization methods that mitigate one of the most critical barriers to widespread adoption of wood in safety-sensitive applications. Building on this foundation, this review examines the three domains of processing routes, fire performance, and multifunctional applications in an integrated manner, with particular emphasis on clarifying their direct interconnections. Figure 1 provides an overview of the relationships among wood processing methods, fire performance, and multifunctional applications. Additionally, it explores the rapidly growing landscape of multifunctional engineered wood, emphasizing its applications in areas such as energy storage, conversion devices, structural applications, environmental remediation and water treatment. Finally, this review highlights the remaining challenges and outlines key future directions that will shape the development of next-generation wood-based materials.

## 2. Review Methodology

**Figure 2:**
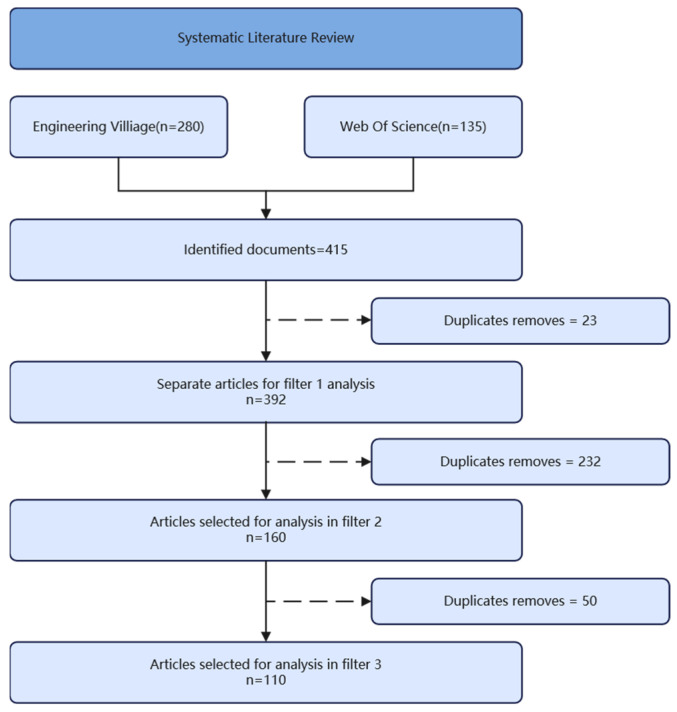
Flowchart for each filtering stage.

This review applies a systematic protocol to capture recent advances in flame-retardant functionalization of wood. Literature was retrieved from major databases between 2016 and 2025, with earlier studies considered only for foundational context. As shown in Figure 2, the initial search identified 415 documents, from which 23 duplicates were removed, resulting in 392 articles for the first-stage screening. Subsequent filtering was conducted in two phases based on predefined criteria. In the second stage, 232 articles were excluded for not meeting the core focus of the review, including those involving non-wood substrates, lacking flame-retardant functionality, missing wood processing or functionalization processes, employing conventional flame-retardant treatments without innovation, or failing to integrate multiple functions. This yielded 160 articles for further evaluation.

**Figure 3:**
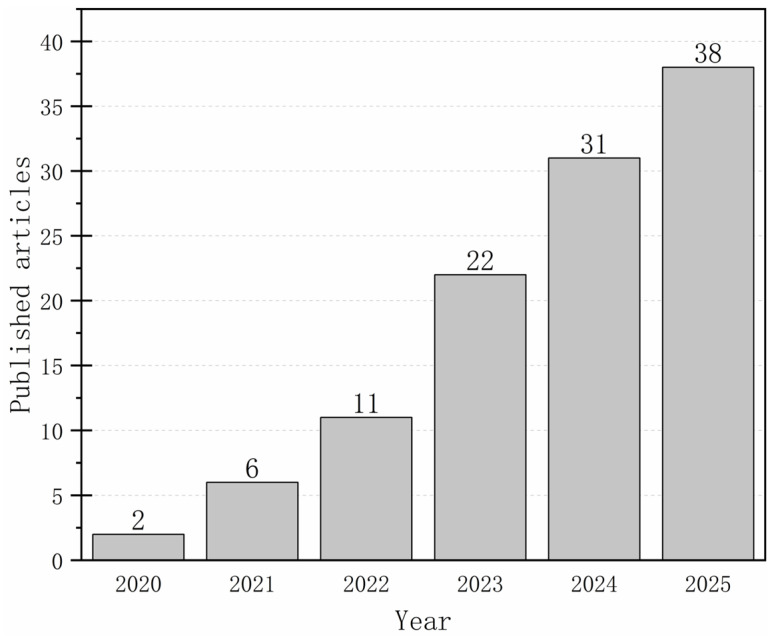
Year of publication of selected publications.

In the third screening stage, 50 articles were excluded because they lacked experimental data, involved unclear or halogen-based flame-retardant systems, were published only as conference abstracts, or appeared in lower-quality journals. The final dataset comprised 110 high-quality original research papers that presented novel modification strategies, reported standardized fire safety metrics, and included supporting structural characterization. Reviews, patents, and conventional treatments without innovative mechanisms were systematically left out. As shown in Figure 3, the number of studies on flame-retardant wood has exhibited a steadily increasing trend over the years.

The review was structured around three core domains: wood processing technologies, flame-retardant functionalization strategies, and multifunctional applications. Special emphasis was placed on identifying interrelationships among processing routes, fire performance, and functional outcomes. Data extraction focused on material composition, processing methods, flame-retardant performance, mechanical properties, and application potential. Tables and figures were used to synthesize comparative performance across different modification strategies, highlighting both advantages and limitations.

To ensure comprehensiveness and relevance, the literature search was conducted using keywords including “wood,” “flame retardant”, “processing”, “application”, and related terms. Only peer-reviewed articles published in English were considered. The methodology aimed to provide a balanced and critical evaluation of current technologies while identifying emerging trends and research gaps for future investigation.

## 3. The Hierarchical Structure and Composition of Natural Wood

At the macroscopic scale, wood exhibits strong anisotropy, with its mechanical, thermal, and transport properties (i.e., the movement of fluids, heat, and chemicals) differing along the longitudinal, radial, and tangential directions [49,50]. This directional behavior arises from its highly organized cellular structure. Wood exhibits a relatively low pyrolysis temperature, with thermal decomposition proceeding sequentially according to the stability of its constituent biopolymers [51]. The process begins with hemicellulose, which decomposes between 180 and 350 °C, followed by cellulose at 275–350 °C and lignin over a broader range of 250–500 °C [52]. These decomposition temperatures make wood inherently susceptible to ignition under fire conditions. Hemicellulose, the most thermally sensitive component, undergoes two-stage degradation: it first breaks down into soluble oligomeric fragments and monomer units, which subsequently decompose into volatile products [53]. This early-stage degradation accelerates pyrolysis and generates combustible gases that can further enhance flame propagation. As decomposition progresses, wood combustion produces smoke and a variety of hazardous fumes, including carbon monoxide, volatile organic compounds, and particulate matter. Exposure to these emissions poses significant risks to human health, potentially causing respiratory distress, eye irritation, nausea, vomiting, and asphyxiation or death. Understanding the pyrolytic behavior and toxic emissions of wood is therefore essential for developing effective flame-retardant treatments and designing materials that minimize fire hazards while ensuring human safety.

## 4. Foundational Processing for Functionalization

Recent innovations in wood processing have transformed a traditional, naturally heterogeneous material into a high-performance, engineered substrate with tunable properties. Modern techniques focus on manipulating the hierarchical structure of wood at micro- and nanoscale levels to enhance mechanical strength, dimensional stability, and functional versatility [54]. Among the key processing strategies, delignification removes lignin while maintaining the cellulose and hemicellulose frameworks, yielding lightweight, porous scaffolds that can subsequently undergo densification, infusion, or chemical modification. Densification, typically achieved through hot pressing or resin impregnation, enhances density and hardness while reducing porosity, producing materials with mechanical strength and stiffness comparable to synthetic composites. In addition, surface treatments and chemical modifications such as esterification, acetylation, and the grafting of functional polymers allow precise adjustment of hydrophobicity, adhesion, and interfacial compatibility with other materials.

### 4.1. Delignification and Densification

A transformative strategy in wood engineering centers on the selective removal of lignin and hemicellulose, the matrix polymers that provide cohesion and rigidity within the cell wall [55]. This process is typically accomplished through chemical treatments employing solutions such as acetic acid combined with hydrogen peroxide or sodium hydroxide with sulfites. By partially or completely eliminating these components, the natural hierarchical architecture of wood is opened, producing a scaffold composed predominantly of aligned cellulose nanofibrils. This porous framework serves as an ideal precursor for densification, where hot pressing collapses the void spaces, resulting in a substantial increase in density and mechanical robustness. The final material, commonly known as densified wood or nanowood, demonstrates exceptional properties, including significantly enhanced tensile strength, markedly increased hardness, and superior dimensional stability under varying environmental conditions. Beyond these improvements, the aligned cellulose fibrils impart anisotropic thermal and mechanical behavior, which can be exploited for directional heat management, energy storage, and other multifunctional applications. By transforming the naturally occurring wood structure into a dense, high-performance material, this approach bridges the gap between renewable biomass and advanced engineered materials, enabling wood to meet the demands of modern structural and multifunctional technologies.

**Figure 4:**
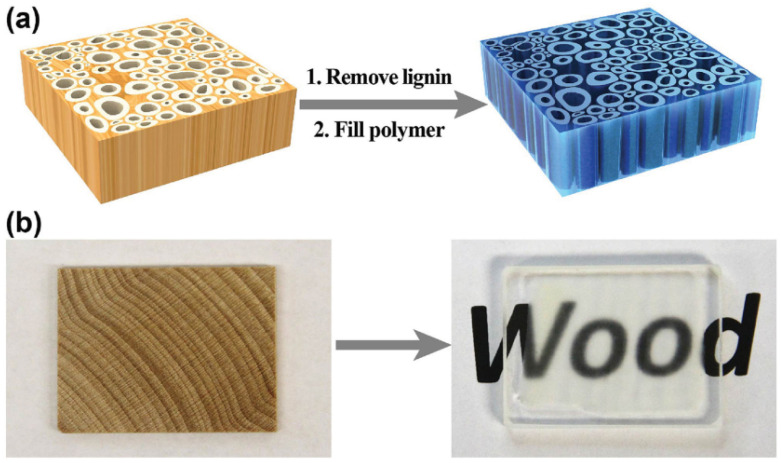
(**a**) Schematic to display the mesoporous structures in wood where the cell walls are aligned vertically. After lignin is removed and the index-matching polymer is filled in, the thick (up to centimeter) piece of wood becomes a highly transparent structural material. (**b**) Pictures to show that wood becomes highly transparent after the two steps [56]. Copyright 2016. Reproduced with permission from WILEY-VCH.

As depicted in Figure 4, Hu et al. [56] prepared highly transparent wood composites by first carrying out a delignification process followed by polymer infiltration. Lignin, which is the colored component responsible for wood’s opacity, was removed using a two-step chemical treatment with sodium hydroxide and sodium sulfite followed by hydrogen peroxide bleaching. This process effectively eliminated lignin while preserving the hierarchical cellulose framework and aligned channel structures of the wood. The efficiency of delignification was found to depend on the cutting direction, with radially cut wood allowing faster lignin removal compared to longitudinally cut wood due to shorter lumina. After delignification, the porous white scaffold was impregnated with a refractive index-matching epoxy resin, which took over the binding function of lignin while maintaining the natural anisotropy of the structure. As a result, the composites achieved up to 90% optical transmittance, strong light scattering, and enhanced mechanical performance.

### 4.2. Wood-Derived Nanocellulose

Wood-derived nanocellulose has emerged as a highly promising sustainable nanomaterial, attracting considerable attention in both scientific research and industrial applications [57,58,59,60,61,62]. As the most abundant natural polymer, cellulose is a primary structural component of wood, organized hierarchically into microfibrils embedded within a lignin–hemicellulose matrix. Through controlled chemical, enzymatic, or mechanical treatments, these microfibrils can be extracted and converted into nanocellulose with dimensions typically in the nanometer range. This nanomaterial exhibits exceptional mechanical strength, high surface area, tunable surface chemistry, and biodegradability, making it an ideal candidate for reinforcing polymers, fabricating advanced composites, and developing functional coatings. Its renewable origin and environmental compatibility further enhance its appeal for sustainable material design, enabling applications across packaging, biomedicine, electronics, and flame-retardant materials.

Fu et al. [63] developed a multifunctional nanostructured wood composite combining flame retardancy, electromagnetic shielding, and photothermal conversion. They used delignified wood with a three-dimensional porous structure as a scaffold for in situ polymerization of polyaniline in an H_3_PO_4_ acidic medium. Removal of lignin and hemicellulose yielded a cellulose-rich aerogel with a hierarchical porous network that allowed uniform polyaniline coating. The resulting polyaniline-wood aerogel exhibited high conductivity of 22.07 S/m and an electromagnetic interference shielding effectiveness of 27.63 dB in the X band at a thickness of 2–3 mm with only 12.5% aniline monomer. The material also showed excellent flame retardancy, preserving its shape after repeated ignition, and efficient photothermal conversion under near-infrared irradiation. The exposed cellulose nanofibers and anisotropic microchannels facilitated polyaniline cross-linking, forming a stable conductive network and highlighting its potential as a renewable, lightweight, and environmentally friendly electromagnetic shielding material.

### 4.3. Implications for Functionalization

Advances in wood processing are reshaping both the mechanical and structural characteristics of this long-utilized material while simultaneously opening new avenues for functionalization. Through delignification and densification, cellulose-rich scaffolds with adjustable porosity and anisotropy can be generated, offering adaptable platforms for chemical modification, polymer infiltration, and hybridization with nanoscale building blocks. Such engineered architectures enable wood to move beyond the role of a structural material, integrating attributes such as optical transparency, directional heat management, and enhanced flame resistance. lignin removal generates a porous scaffold that provides an ideal pathway for deep and uniform impregnation of flame retardants, overcoming the uneven distribution typically observed in untreated wood [64,65]. Control over the hierarchical organization from the molecular to the macroscale establishes a direct pathway to multifunctional composites capable of matching or even exceeding synthetic alternatives.

The development of wood-derived nanocellulose provides an equally transformative direction. With its high aspect ratio, large surface area, and chemically tunable interfaces, nanocellulose facilitates the reinforcement of polymers, the fabrication of responsive coatings, and the construction of biocompatible systems. Its renewable origin and biodegradability further support the sustainable design of advanced materials for applications ranging from packaging and biomedicine to electronics and energy storage. When combined with conductive polymers, as demonstrated by polyaniline–wood hybrids, these frameworks extend functionality to electromagnetic shielding and photothermal conversion, highlighting the adaptability of wood-based nanostructures. Together, these advances demonstrate how processing-driven control of hierarchical frameworks can act as a universal strategy for embedding multifunctionality, positioning wood as a tunable and sustainable substrate for next-generation technologies.

## 5. Flame-Retardant Functionalization of Wood: Mechanisms, Applicability, and Performance Optimization

Flame-retardant functionalization of wood has become a pivotal strategy to enhance the fire safety of wood-based materials while preserving their structural integrity and esthetic qualities [66,67,68]. As a natural and renewable polymeric material, wood is inherently flammable because cellulose, hemicellulose, and lignin readily undergo thermal degradation, releasing combustible volatiles. To overcome this limitation, a variety of chemical and physical modification strategies have been developed, including impregnation with phosphorus-, nitrogen-, or silicon-based flame retardants, surface coating with intumescent or hybrid nanomaterials, and in situ polymerization of protective or conductive polymers. These approaches function by promoting char formation, suppressing heat release, and limiting the emission of smoke and toxic gases during combustion [69,70]. Furthermore, advanced processing techniques, such as delignification and the engineering of hierarchical porous wood structures, enhance the uniform distribution and anchoring of flame-retardant agents, simultaneously improving fire resistance and maintaining mechanical performance. Together, these strategies provide a versatile platform for optimizing the effectiveness, durability, and applicability of flame-retardant wood in construction, protective materials, and multifunctional applications.

### 5.1. Mechanisms of Flame Retardancy in Wood

The flame retardancy of wood is achieved through mechanisms that act in both the condensed and gas phases [42]. In the condensed phase, flame retardants promote the formation of a protective char layer on the wood surface, which insulates the underlying material, slows heat transfer, and limits the release of combustible volatiles. In the gas phase, certain flame-retardant additives release non-combustible gases that dilute flammable pyrolysis products and inhibit radical reactions in the flame. Intumescent systems further enhance fire resistance by forming an expanded, foamed char layer through the combined action of an acid source, a carbon source, and a blowing agent, providing both thermal insulation and physical shielding. Synergistic combinations of phosphorus, nitrogen, silicon, or metal-based compounds can improve char stability, reduce smoke emission, and enhance overall flame retardancy [71]. These mechanisms form the basis for developing effective wood treatments that enhance fire safety while maintaining mechanical integrity.

### 5.2. Bulk Modification Strategies: Penetrating the Hierarchical Structure

Impregnation represents a central approach for enhancing the flame-retardant performance of wood by introducing functional additives or flame-retardant compounds directly into its cellular architecture [72,73,74]. During this process, the additives infiltrate the internal pores, lumina, and microchannels of the wood, acting as fillers that improve thermal stability, while chemical modification promotes the formation of covalent bonds with cellulose, hemicellulose, and lignin in the cell walls. This combination of physical filling and chemical integration not only elevates flame resistance but also enhances mechanical strength, dimensional stability, and long-term durability. By fully leveraging the hierarchical structure of wood, bulk modification ensures uniform distribution and intimate contact between modifiers and the wood matrix, maximizing performance. As a result, impregnated and chemically modified wood can be applied across a broad spectrum of structural and functional uses, including load-bearing supports, engineered panels, and composite boards, where both fire safety and mechanical reliability are essential.

#### 5.2.1. Impregnation with Inorganic Additives

Impregnation with inorganic additives has been widely explored as a strategy to improve the performance and service life of wood, particularly in applications requiring enhanced fire safety and environmental durability. During this process, inorganic compounds such as phosphates, borates, silicates, and metal oxides infiltrate the porous cellular structure of wood, where they occupy lumens and partially diffuse into cell walls, thereby altering the physicochemical properties of the lignocellulosic matrix. These additives play a crucial role in modifying the thermal degradation pathway of wood by catalyzing dehydration, facilitating char formation, and generating protective mineral layers that reduce the evolution of combustible volatiles and inhibit heat and mass transfer. Beyond imparting flame retardancy, inorganic impregnation has been shown to decrease hygroscopicity, improve dimensional stability, smoke suppression and provide resistance against biological degradation [75].

The flammability of wood restricts its practical use, but impregnation with inorganic additives provides an effective means of enhancing its fire safety. Chen et al. [76] developed a phytic acid-silica hybrid system in wood via vacuum-pressure impregnation, achieving uniform incorporation of flame-retardant species within the cellular structure. This treatment greatly improved performance, increasing char yield to 32.1%, raising the limiting oxygen index to 47.3%, and reducing both heat release and smoke production. The enhanced flame retardancy and smoke suppression were attributed to the formation of compact, crosslinked char layers that served as protective barriers, while the mechanical properties of wood remained essentially unchanged. When assessing the flame-retardant performance of wood and its composites, the limiting oxygen index (LOI) and vertical burning test (UL-94) are two core methods commonly applied during the laboratory research and development stage. LOI testing, conducted according to standards such as GB/T 2406.2-2009 or ASTM D2863, provides a rapid and quantitative measure of flame-retardant effectiveness by determining the minimum oxygen concentration needed to sustain combustion. In general, a higher LOI value corresponds to stronger resistance to ignition. UL-94 testing, for instance following ASTM D3801-10, classifies materials into categories including V-0, V-1, and V-2 according to vertical burning characteristics such as after-flame duration and the presence of molten dripping, thereby offering a visual indication of the self-extinguishing capacity of the material. Table 1 summarizes the flame-retardant properties of wood treated via various methods, including impregnation, in situ mineralization, and reactive grafting. The data demonstrates that these treatments can significantly enhance the fire resistance of wood, with several formulations achieving the highest UL-94 classification of V-0.

#### 5.2.2. In Situ Mineralization

In situ mineralization has emerged as a powerful strategy for engineering multifunctional wood with enhanced performance. By inducing the formation of nanoscale mineral phases directly within the hierarchical pores and cell walls of wood, this approach enables uniform integration of inorganic components without compromising the natural architecture or appearance. Such mineralized structures can simultaneously improve flame retardancy, thermal stability, and mechanical integrity, while providing additional functionalities including smoke suppression, mold resistance, and antitermite activity. The synergy between the mineral phase and the organic wood matrix promotes the formation of dense char during combustion and impedes the release of flammable volatiles, offering an intrinsic, bulk-level approach to wood functionalization.

Ou et al. [80] developed an eco-friendly impregnation approach combined with in situ mineralization to fabricate multifunctional wood exhibiting enhanced flame retardancy, smoke suppression, mold resistance, and antitermite properties. Nanoscale zinc borate (ZnB) particles were incorporated into the hierarchical porous structure of wood, leading to an increase in the limiting oxygen index from 22.6% to 41.2% and marked reductions in heat release and smoke generation. Upon combustion, the molten ZnB formed a noncombustible layer that reinforced the carbonaceous char, generating a cohesive three-dimensional network that inhibited oxygen penetration, provided thermal insulation, and suppressed toxic smoke. Additionally, ZnB imparted outstanding antitermite and antifungal performance. This method provides a scalable, efficient, and environmentally friendly route to produce bulk-treated, multifunctional wood while retaining its natural appearance, with strong potential for sustainable building applications.

#### 5.2.3. Reactive Grafting and Covalent Bonding

Reactive grafting and covalent bonding have become powerful strategies for precisely modifying the chemical and functional characteristics of wood. By establishing stable covalent connections between functional modifiers and the hydroxyl groups within wood cell walls, these methods allow for the durable incorporation of flame retardants, hydrophobic agents, or other performance-enhancing compounds. Such chemical modifications enhance thermal stability, flame retardancy, and moisture resistance while maintaining the hierarchical structure and mechanical integrity of the native wood. The resulting materials integrate the intrinsic advantages of wood with customized surface and bulk properties, providing a versatile and scalable approach to producing high-performance, multifunctional wood for construction, furniture, and advanced material applications.

Liu et al. [82] proposed a sustainable impregnation method to enhance the flame retardancy and dimensional stability of fast-growing poplar, which is otherwise limited by high flammability and poor stability. In this approach, poplar was treated with a bio-based solution of furfuryl alcohol (FA) and ammonium phytate (AMP), followed by in situ polymerization of FA to immobilize AMP within the wood cells. Thermal analysis revealed that the residual char of AMP/FA-wood at 800 °C increased by 305% compared with untreated poplar. The modified wood exhibited superior flame-retardant performance, achieving a UL-94 V-0 rating and a limiting oxygen index of 36.3%, while substantially reducing heat release and smoke production. The improvements were attributed to the nitrogen-phosphorus synergistic intumescent action of AMP and the high carbon content of polymerized FA, which facilitated dense char formation. Furthermore, chemical bonding between the PFA resin and wood cells enhanced dimensional stability, hardness, and water resistance. This study demonstrates that impregnation with bio-based FA and AMP is an effective, environmentally friendly strategy to produce fast-growing wood with outstanding flame retardancy, thermal stability, and mechanical properties, suitable for furniture, construction, and other applications.

### 5.3. Surface Engineering Strategies

Surface coating represents an effective strategy to enhance the flame retardancy, weather resistance, and mechanical performance of wood while preserving its bulk properties [83,84]. These approaches rely on the deposition of protective layers that act as physical barriers, slow heat transfer, or release flame-inhibiting species during combustion. Common techniques include dip coating, which immerses wood in a coating solution; spray coating, which ensures uniform coverage over complex geometries; layer-by-layer (LbL) assembly, which builds nanostructured multilayers through electrostatic interactions; sol–gel coating, which produces inorganic–organic hybrid films; and electrophoretic deposition, which employs an electric field to drive charged particles onto the wood surface. By carefully selecting coating materials and deposition methods, wood surfaces can be engineered to achieve improved fire resistance, enhanced durability, and additional functional properties, providing a versatile platform for advanced applications without compromising the structural integrity of the underlying material.

#### 5.3.1. Dip Coating Techniques

Dip coating is a widely used surface treatment method for enhancing the flame retardancy, durability, and functional properties of wood [85]. In this technique, wood substrates are immersed in a liquid coating solution containing flame-retardant compounds, polymers, or nanoparticles, allowing the solution to penetrate the surface pores and form a uniform layer upon withdrawal. After dipping, the coated wood is typically dried or cured to stabilize the protective layer. Dip coating is valued for its simplicity, scalability, and ability to uniformly treat complex wood geometries, making it a versatile approach for developing flame-retardant and multifunctional wood materials.

**Figure 5:**
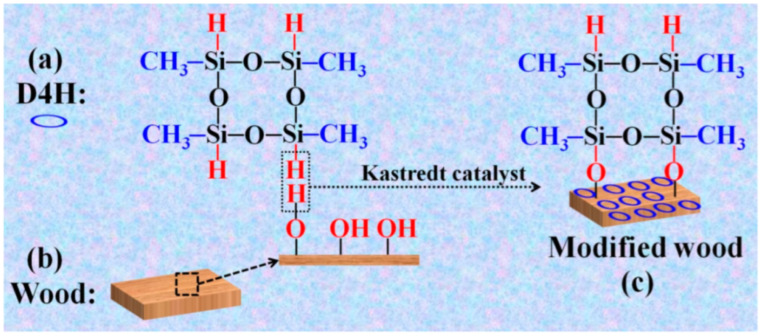
Schematic representation of the process for obtaining the D4H modified wood: (**a**) D4H structure; (**b**) Wood; (**c**) Modified wood [86].

Cai et al. [86] developed a simple and effective dip coating strategy to fabricate hydrophobic and anti-fouling surfaces on wood substrates. As shown in Figure 5, wood samples were immersed in a tetramethylcyclotetrasiloxane (D4H) solution for 5 min, enabling the low-surface-energy D4H to covalently graft onto the wood surface. The treated wood exhibited excellent hydrophobicity, with water contact angles of 140.1° and 152° on the radial and cross sections, respectively, and reduced water absorption (<40% after 24 h). Additionally, the D4H-coated wood demonstrated strong anti-fouling performance, UV resistance, and mechanical durability under tape peel and finger-wiping tests.

#### 5.3.2. Spray Coating Methods

Spray coating is a widely used technique for enhancing the flame retardancy and functional properties of wood [87]. In this method, a protective solution is atomized into fine droplets and applied uniformly to the wood surface, allowing precise control over coating thickness and coverage, even on irregular geometries. Common coatings used in flame-retardant spray applications include epoxy, acrylic, and polyurethane resins. Among these, epoxy resins provide the strongest adhesion to wood, ensuring durable and stable coatings, while acrylic and polyurethane resins contribute flexibility, weather resistance, and compatibility with various flame-retardant additives. By optimizing spraying parameters such as pressure, nozzle type, and solution viscosity, high-performance flame-retardant wood surfaces can be achieved efficiently.

**Figure 6:**
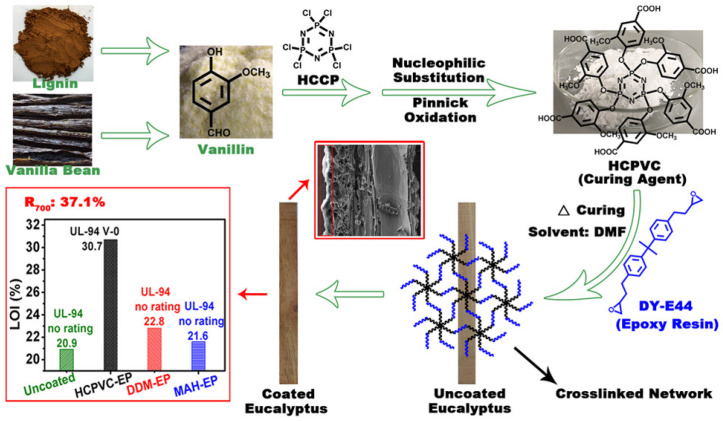
Mechanism diagram of HCPVC curing epoxy resin on the wood surface [88]. Copyright 2019. Reproduced with permission from American Chemical Society.

Guo et al. [88] reported the use of a novel biobased polybasic carboxylic acid, HCPVC, consisting of vanillin and hexachlorocyclotriphosphazene, as a curing agent in wood epoxy coatings via a dip coating process (Figure 6). HCPVC was synthesized through nucleophilic substitution and Pinnick oxidation, and its structure was confirmed by FTIR and ^1^H NMR. Epoxy resin (DY-E44) coatings on eucalyptus wood were cured stepwise with HCPVC under optimized conditions determined by DSC, showing excellent hardness, adhesion, water resistance, and solvent resistance. The HCPVC-cured epoxy (HCPVC-EP) achieved a high char yield of 37.1% at 700 °C, significantly higher than conventional coatings, and exhibited superior flame retardancy with a UL-94 V-0 rating and an LOI of 30.7%.

#### 5.3.3. Layer-by-Layer (LbL) Assembly

LbL assembly is a versatile surface modification technique for wood that enables precise construction of nanoscale multilayer coatings through alternate deposition of oppositely charged polyelectrolytes, nanoparticles, or functional molecules [89,90]. This method allows fine control over coating thickness, composition, and functionality, making it particularly effective for enhancing flame retardancy, hydrophobicity, or other surface properties without significantly altering the bulk structure of the wood. LbL-assembled coatings can form compact, uniform, and conformal layers that improve char formation during combustion and reduce the release of flammable volatiles. The technique is highly adaptable, allowing integration of multiple functional additives to create multifunctional wood surfaces with tunable performance for fire safety, durability, and environmental resistance.

Yan et al. [91] employed a LbL self-assembly strategy to construct intumescent flame-retardant coatings on wood using polyethyleneimine (PEI) and ammonium polyphosphate (APP), followed by cross-linking with divalent metal ions. SEM-EDX and FTIR analyses confirmed the successful deposition of PEI-APP multilayers and their stabilization through Cu^2+^ and Co^2+^ cross-linking. Thermogravimetric analysis showed that the LbL-assembled PEI-APP coatings significantly improved the thermal stability of wood by reducing the maximum degradation rate and increasing char residue, while metal-ion cross-linking further enhanced these effects. The flame-retardant performance was markedly improved, with the limiting oxygen index (LOI) increasing from 23.5 for uncoated wood to 37.5 for PEI-APP coated wood, and further up to 47 and 42.5 after Cu^2+^ and Co^2+^ cross-linking, respectively. Cone calorimeter results revealed notable reductions in peak heat release rate, total heat release, and smoke release, indicating enhanced fire safety. The superior performance was attributed to the synergistic effect of APP and metal ions, which promoted catalytic char formation and generated a stable, expanded carbon layer that effectively shielded the wood from heat and oxygen.

#### 5.3.4. Sol–Gel Coating Approaches

**Figure 7:**
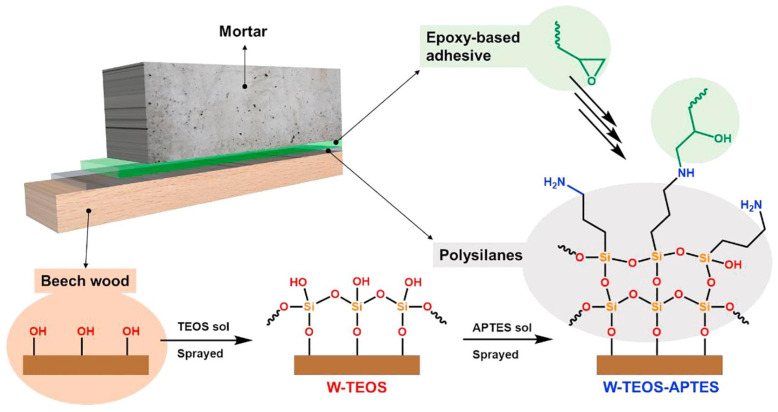
Schematic describing the route to fully glued connections between beech wood and mortar. The surface treatment is based on a two-step sol–gel process, leading to wood coating with polysilanes, which can undergo further reactions with the epoxy functionalities of the glue [92]. Copyright 2018. Reproduced with permission from Elsevier Science Ltd.

Kostic et al. [92] investigated the use of sol–gel coatings to functionalize wood surfaces and enhance adhesion in timber-mortar composite structures. As shown in Figure 7, beech wood was pre-treated with a xerogel obtained via a sol–gel process, consisting of two layers of silane nanofilms, tetraethoxysilane and 3-Aminopropyl triethoxysilane. Chemical analysis confirmed the formation of covalent bonds between the silanes and the wood surface, while microscopy revealed that the coatings influenced adhesive penetration. Mechanical tests showed that the sol–gel treated wood exhibited improved load-bearing capacity in wood-mortar composites compared to untreated specimens. The amine-functionalized silane enhanced interfacial bonding with the epoxy adhesive, and the treatment also improved wettability and interface compatibility. Tsvetkova [93] prepared a “sol–gel@paint” formulation incorporating wax and silicone, which was applied to pine wood for laboratory testing. The inclusion of hydrophobically treated colloidal silica (aerosol) in the sol–gel@paint significantly increased the water contact angle, reaching 110°.

### 5.4. Applicability of Flame-Retardant Strategies

Bulk modification strategies rely fundamentally on the accessibility and permeability of the wood’s internal structure. Impregnation with inorganic additives, including ammonium polyphosphate or borate salts, is most effective in low-density native softwoods and achieves even greater efficiency in delignified wood scaffolds. The selective removal of lignin and hemicellulose generates an open, highly porous network of cellulose nanofibrils, eliminating natural anatomical barriers and enabling the capillary-driven, uniform penetration of aqueous solutions. This facilitates the incorporation of high additive loadings necessary for effective flame retardancy. In situ mineralization is particularly suited to delignified templates, where the hydrophilic, nanofibrillar, and charged surfaces of cellulose act as bio-inspired scaffolds for the nucleation and growth of mineral phases, such as calcium carbonate or hydroxyapatite, converting the organic framework into a homogeneous organic–inorganic composite. The success of this approach is critically dependent on the nanoscale interface. Similarly, reactive grafting and covalent bonding require not only physical but also chemical accessibility; efficacy is maximized on delignified wood, where the extensive removal of lignin exposes a larger population of cellulose hydroxyl groups that serve as anchoring points for phosphorus- or silicon-based flame-retardant moieties. Substrates in which these reactive sites are sterically hindered are less amenable to this strategy.

In contrast, surface engineering strategies target the external interface and can be applied across a wider range of substrates, although performance remains influenced by surface properties. Dip coating is most effective on porous wood capable of absorbing treatment solutions via capillary action, with enhanced results on delignified scaffolds due to increased surface area and wettability, while still providing superficial protection to untreated wood. Spray coating allows uniform coverage of complex geometries and large surfaces, making it especially suitable for engineered products such as cross-laminated timber or curved elements, where immersion is impractical, and relies on the formation of a continuous surface film. Layer-by-layer assembly offers exceptional versatility, enabling nanoscale, conformal coatings on both smooth densified surfaces and the extensive internal surfaces of delignified wood through electrostatic interactions. Charged surfaces, either inherent or introduced via a polyelectrolyte primer, facilitate precise deposition on diverse architectures. Sol–gel coatings are designed to form adherent, ceramic-like layers primarily on exterior surfaces of densified or engineered wood, creating continuous protective barriers. While the precursor sol can partially infiltrate porous woods to form hybrid layers, the principal function remains the formation of a robust, surface-functional coating.

### 5.5. Performance Trade-Offs of Wood Modification Strategies

For bulk modification strategies, the primary trade-off revolves around balancing flame retardancy with mechanical integrity and long-term stability. Impregnation with inorganic additives achieves significant reductions in pHRR and THR but often compromises mechanical plasticity due to crystal formation within cell lumens, leading to embrittlement. Additionally, the inherent hydrophilicity of these salts introduces dimensional instability under humid conditions and promotes leaching, which curtails long-term efficacy, especially in exterior applications. In situ mineralization effectively establishes thermal barriers but frequently leads to increased material brittleness. Studies have reported reductions in strain at failure of 80–90%, accompanied by a decrease in tensile strength from 480 ± 8 MPa to 439 ± 9 MPa [94]. Reactive grafting and covalent bonding provide permanent flame-retardant properties and enhanced dimensional stability but require sophisticated chemical processes that increase processing costs compared to conventional treatments and may compromise the native wood’s viscoelasticity through cross-linking.

Surface engineering strategies generally preserve the bulk mechanical properties of wood but introduce trade-offs in durability, processing complexity, and sometimes optical clarity. Dip coating techniques provide uniform coverage but can add significant mass, potentially limiting structural applications, and thicker coatings may obscure wood’s natural esthetic. Spray coating methods offer rapid application and adaptability to complex geometries but often suffer from inconsistent film formation. LbL assembly achieves exceptional nanoscale control and strong reductions in pHRR and THR, but the deposition process is time-intensive, creating significant scalability challenges for industrial adoption.

**Figure 8:**
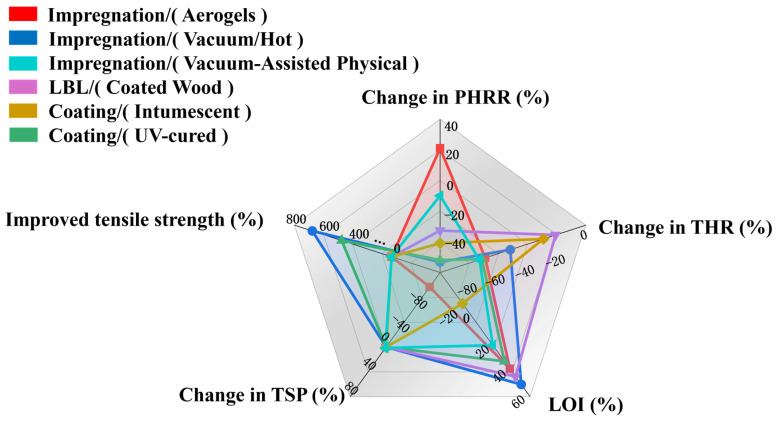
Comparative evaluation of flame-retardant treatments for wood: aerogel-assisted impregnation [95], vacuum/hot-pressing assisted impregnation [96], vacuum-assisted physical impregnation [96], layer-by-layer (LBL) coating [91], intumescent coating [97], and UV-cured coating [98].

Table 2 presents a comparison of different flame-retardant treatments, such as aerogel impregnation, chemical densification, mineralization, and intumescent coatings, in relation to wood’s fire performance, smoke production, and tensile strength. The outcomes show that many of these methods provide marked improvements in fire suppression, while some also enhance mechanical properties, emphasizing their potential for the development of high-performance wood materials. As illustrated in Figure 8, the comparative evaluation of flame-retardant treatments for wood demonstrates clear differences in mechanical, flame-retardant, and smoke-suppressing performances. In terms of mechanical strength, impregnation methods (e.g., vacuum or hot impregnation) deliver the most significant improvement in tensile strength, surpassing both the LBL and intumescent coating techniques. This indicates that impregnation effectively enhances the intrinsic structure of wood. Moreover, the LOI of impregnated wood shows a marked increase. It is noteworthy, however, that certain approaches such as UV-coating and LBL may reduce tensile strength compared to impregnation, suggesting that specific processing parameters strongly influence the final properties. Regarding flame retardancy, intumescent coatings and UV-coatings are particularly effective in reducing the peak heat release rate, followed by the LBL method. This highlights the ability of surface coatings to create protective barriers that slow down flame spread. In terms of smoke suppression, impregnation with aerogels provides a distinct advantage by significantly lowering total smoke production.

**Table 2:** Comprehensive evaluation of flame retardancy, smoke suppression, and mechanical properties of wood treated with various advanced methods.

Wood	Flame Retardant Methods	Fillers Content (wt%)	PHRR (KW/m^2^)	THR (MJ/m^2^)	LOI (%)	TSP (m^2^)	Tensile Strength (MPa)	Building-Code Taxonomy	Ref.
LDH-PANI-wood (PANI-LDH/MgAl)	Impregnation of nanofibrous flame-retardant aerogels	16 wt% LDH + 11 wt% PANI	272 (+21.1%)	0.54 (−69%)	42	0.14 (−96.69%)	N/A	N/A	[95]
ADP-DW_1_ (ADP)	Hot-pressing Assisted Chemical Impregnation	2.0 mol/L ADP	92.9 (−53.1%)	29.2 (−51.8%)	52.1	N/A	325.4 (+653%)	Cone calorimeter: ASTM (2017); LOI: GB/T 2406.2-2009	[96]
DW_2_/PA_4_ (PA)	Ambient Pressure Impregnation	4 wt% PA	96.4 (−51.89%)	14.2 (−71.37%)	37.2	N/A	138 (+411%)	N/A	[99]
FRTW-wood (PAA)	Vacuum-Assisted Polymer Precursor Impregnation	15 wt% PAA	53.9 (−56.59%)	8.6 (−27.37%)	N/A	N/A	169 (+4125%)	N/A	[100]
WL-wood (BN)	Hot-pressing Assisted Chemical Impregnation	6 wt% BN	175.2 (−50.1%)	9.8 (−43.68%)	N/A	N/A	330 (+864%)	N/A	[101]
MW(ZnB)	Vacuum-Assisted Impregnation	22.1%ZnB	58 (−46.9%)	36.5 (−47.9%)	41.2	0.13 (−81.3%)	N/A	LOI: ASTM D2863-2017; Cone calorimeter: ISO 5660-1(2002)	[80]
(PEI-APP)_15_ -CW (PEI/APP/Cu^2+^/Co^2+^)	layer-by-layer (LBL)	1 wt% PEI + 1 wt% + 1 mol/L (Cu^2+^/Co^2+^)	128.87 (−32.6%)	61.4 (−20.7%)	47	N/A	N/A	LOI: GB/T 2406.2-2009	[91]
TW/PEAG-wood (PA/PER/PEG/MF)	Vacuum-Assisted Impregnation	PEAG:MF = 60:120 g	178.3 (−82.4%)	9.8 (−84.3%)	37	N/A	153.6 (+500%)	Cone Calorimeter: ASTME1354	[102]
CW(CO-SH/DA/TTC)	UV-cured coating	CO-SH:DA:TTC = 1:0.6:0.6	147.63 (−9.9%)	16.99 (−72.95%)	26.83	2.01 (+1.5%)	N/A	LOI: ASTM D2863; UL-94: ASTM D3801	[98]
MPEA-wood (PEA/MPEAs/MF)	Intumescent fire-retardant coating	PEA:MH = 95:5	95.9 (−40.9%)	2.2 (−29%)	N/A	N/A	N/A	N/A	[97]
Steel-EP/9P -MT-HOF -wood (MA/TPA/PA)	Intumescent fire-retardant coating	9 wt% P-MT-HOF	380 (−61.6%)	50 (−32.9%)	30.3	17 (−32.1%)	79.2 (+39.3%)	N/A	[103]

Note: The abbreviations used in the table are defined as follows: N/A indicates data that is not available. Terms related to material types include FRTW (flame-retardant transparent wood), CW (coated wood), DW_1_ (densified wood), DW_2_ (delignified wood), TW (transparent wood), WL (Wood Laminateand), and MW (mineralized wood). Chemical compounds and treatments are abbreviated as: LDH-PANI (layered double hydroxide-polyaniline), ADP (ammonium dihydrogen phosphate), PA (phytic acid, or phosphoric acid/pyrophosphoric acid as context-dependent), PAA (polyamide acid), PEI (polyethyleneimine), APP (ammonium polyphosphate), PEAG (phosphate ester-polyethylene glycol), PER (pentaerythritol), PEG (polyethylene glycol), MF (melamine formaldehyde/resin), CO-SH (castor oil-grafted thiol), BN (bentonite nanosheets), DA (reaction product of DDP and AGE), TTC (2,4,6-trimethyl-2,4,6-trivinylcyclotrisilazane), PEA (phosphate ester), MPEA (magnesium phosphate ester), EP (epoxy resin), P-MT-HOF (pyrophosphoric acid-grafted melamine-terephthalic acid hydrogen-bonded organic framework), MA (melamine), and TPA (terephthalic acid).

In conclusion, the selection of a flame-retardant strategy for wood involves navigating a multi-dimensional trade-off space. No single approach is universally superior; the optimal choice depends on the specific application requirements, balancing fire safety, mechanical performance, economic constraints, and environmental sustainability. Future research is increasingly focused on developing hybrid and bio-based solutions that minimize these compromises through innovative chemistry and processing techniques

## 6. Various Applications of Wood

### 6.1. Structural Applications

Wood has served as a primary structural material for millennia, forming the backbone of traditional architecture [104,105,106,107]. Its widespread replacement by man-made materials such as steel and concrete has largely been driven by limitations in mechanical performance. Table 3 presents an overview of various multifunctional materials, highlighting their synthesis processes and potential applications beyond conventional use. Nevertheless, from a sustainability perspective, wood offers a markedly lower environmental footprint, requiring minimal industrial processing compared with synthetic alternatives. Recent advances reveal that highly crystalline cellulose nanofibrils extracted from wood exhibit exceptional mechanical properties, with Young’s modulus in the range of 120–220 GPa and tensile strength of 1.6–3 GPa, comparable to multiwalled carbon nanotubes and synthetic Kevlar fibers [108,109,110,111].

Building on these intrinsic properties, engineered wood products including cross-laminated timber (CLT), laminated veneer lumber (LVL), and glue-laminated timber (glulam) enable the construction of large-scale structures with enhanced strength, stiffness, and dimensional stability. Furthermore, the integration of nanocellulose and other wood-derived nanomaterials into composite architectures allows the development of lightweight, high-performance materials that rival conventional metals and polymers. Such hybrid materials not only leverage the hierarchical organization of cellulose fibers but also offer tunable mechanical, thermal, and acoustic properties, opening pathways for applications in tall buildings, bridges, and load-bearing components. Collectively, these advances highlight the potential of wood-based materials as sustainable, high-performance alternatives for modern structural engineering, combining ecological benefits with unprecedented mechanical functionality.

The inherent dimensional instability and flammability of wood have traditionally limited its use in structural applications. Guo et al. [112] demonstrated that combining surface densification with targeted chemical modifications can produce wood with exceptional mechanical performance, dimensional stability, and flame retardancy. The resulting material exhibits a flexural strength of 193.98 MPa, surpassing most conventional inorganic and organic materials, and achieves a strength-to-weight ratio 4.4 times higher than that of 1040 alloy steel. Water immersion tests further highlight its stability, with a rebound rate of only 2.62% and water absorption of 3.16% after 24 h, remaining below 20% even after six days. Surface densification primarily enhances mechanical strength, while incorporation of ammonium dihydrogen phosphate (ADP) into cell walls improves flame retardancy and dimensional stability, though at some cost to mechanical properties. This reduction is effectively counteracted by introducing epoxy polymer into the cell lumens, restoring strength and further stabilizing dimensions. Collectively, this multifunctional treatment addresses the traditional limitations of wood, rendering it a viable candidate for high-performance structural applications.

**Table 3:** Fabrication approaches and functional applications of wood-based composites.

Key Raw Materials	Fabrication	Application	Advantage	Predominant Properties	Ref
APP/TA/Silica sol	Vacuum Pressure Impregnation	Traditional wooden architecture	Excellent collaborative flame retardancy	Mechanical properties	[113]
GO/BA	Graded-Penetration Impregnation, Hot-Pressing	Fire alarm	Integrated flame-retardant, fire-warning	Anti-microbial, mechanical enhancement	[114]
C-MOFs@ACW	Carbonization of wood	Energy storage	Superior energy density	Enhanced supercapacitor performance and long-term cycle life	[115]
PdCl_2_/NaBH_4_/MB/MO/4-NP	Immersion	Water treatment	Selective high-efficiency degradation	Sustainability	[116]
PDMS/SiO_2_/ETA-APP/PER	Distribution coating	Environmental protections	Self-cleaning, weather-resistant	Durability and environmental adaptability	[29]
Carbon Nanoparticles	Flame Treatment	Solar Steam Generation	Low cost, high light absorption rate, hydrophilic, Environmental sustainability	Thermal insulation property and carbon layer has strong adhesion	[117]

Note: The abbreviations used in the table are defined as follows: N/A indicates data that is not available; APP, ammonium polyphosphate; TA, tannic acid; GO, graphene oxide; BA, boric acid; MOFs, metal–organic frameworks; C-MOFs@ACW, carbonized/activated MOFs@Wood derived hierarchical porous composites; MB, Methylene Blue; MO, Methylene Orange; 4-NP, 4-Nitrophenol; PDMS, poly(dimethylsiloxane); ETA-APP, ethanolamine-modified ammonium polyphosphate; PER, pentaerythritol; TWF, transparent wood film; LCF, lignin-derived carbon fibers.

### 6.2. Environmental Remediation and Water Treatment

Beyond traditional structural roles, wood has emerged as a versatile material for environmental remediation and water treatment [118,119,120,121]. Its naturally porous architecture and abundant hydroxyl groups provide a high surface area and rich chemical functionality, enabling efficient adsorption of pollutants including heavy metals, dyes, and organic contaminants. Recent studies have demonstrated that chemically modified wood and wood-derived nanocellulose can selectively capture toxic species from water while maintaining structural integrity and regenerability.

**Figure 9:**
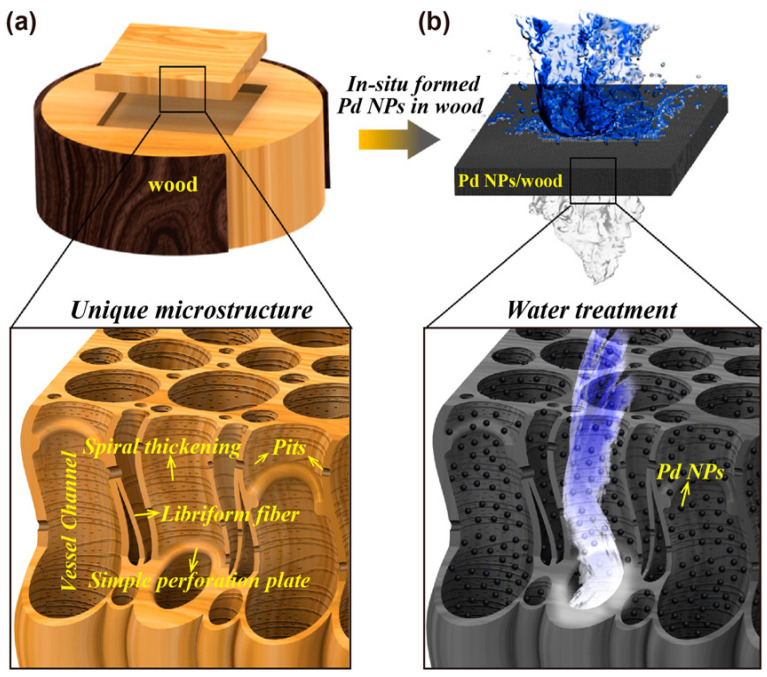
Basswood decorated with Pd NPs for water treatment. (**a**) Schematic of a 3D wood membrane decorated with Pd NPs for water treatment. (**b**) In situ formed Pd NPs within the wood where lignin acts as the reducing agent [122]. Copyright 2017. Reproduced with permission from American Chemical Society.

As depicted in Figure 9, Hu et al. [122] fabricated a mesoporous three-dimensional wood membrane decorated with palladium nanoparticles (Pd NPs/wood membrane) for efficient wastewater treatment. Within the wood mesostructure, lignin reduces Pd(II) ions to Pd NPs, while cellulose hydroxyl groups stabilize and immobilize them. The partially aligned channels and complex microstructures enhance contact between wastewater and Pd NPs, and the long Pd NP-decorated channels enable continuous treatment during water flow. As a demonstration, the Pd NPs/wood membrane achieved rapid and highly efficient methylene blue removal, with a turnover frequency of ~2.02 mol_MB_·mol_Pd_^−1^·min^−1^, far exceeding reported values. In addition, it showed an ultrahigh treatment rate of 1 × 10^5^ L·m^−2^·h^−1^ with removal efficiency above 99.8%.

Effective dehydration of water-in-crude oil emulsions is critical for reducing transportation costs, energy consumption, and pipeline corrosion. Greiner et al. [123] reported carboxylated wood-based sponges (CWS) derived from delignified and hemicellulose-removed balsa wood, which possess aligned cellulose nanofibers and a lamellar architecture. This structure imparts high mechanical compressibility, excellent elastic recovery, and underoil superhydrophilicity. The CWS demonstrate remarkable water absorption, with a capacity of approximately 15 g g^−1^, and achieve dehydration efficiencies exceeding 99.99%, reducing water residuals in crude oil to as low as 20 ppm. The absorbed crude oil can be readily recovered through simple squeezing, and the sponges maintain performance over long-term cycles without degradation. Moreover, the CWS exhibit robust chemical stability under extreme acidic and basic conditions. These findings highlight the potential of carboxylated wood-based sponges as highly effective, reusable, and chemically resilient materials for industrial-scale deep dehydration of crude oil and other water-in-oil separations.

Nanocellulose has emerged as a highly effective bio-absorbent due to its versatile surface chemistry and tunable macroscopic forms, which facilitate post-treatment recovery and regeneration. Among these, cellulose aerogels with three-dimensional interconnected networks and intact physical structures have been widely employed for wastewater treatment. Hsieh et al. [124] reported the fabrication of ultra-light and ultra-porous cellulose nanofibril (CNF) aerogels derived from rice straw cellulose with a defibrillation yield of 96.8%. These aerogels, with densities ranging from 1.7 to 8.1 mg cm^−3^ and porosities of 99.5 to 99.9%, exhibit amphiphilic super-absorbent properties, absorbing up to 210 times their weight in water and 375 times in chloroform, surpassing previously reported cellulose aerogels. Chemical vapor deposition of triethoxyl(octyl) silane further enhanced their hydrophobicity and oleophilicity, allowing absorption of 139 to 356 times their weight in non-polar hydrocarbons, polar aprotic solvents, and oils, exceeding the performance of polymeric, cellulosic, and carbonaceous aerogels by up to 20-fold. The aerogels also demonstrate excellent mechanical resilience, maintaining superior wet compressibility and complete shape recovery in water over 100 cycles.

### 6.3. Energy Storage and Conversion Devices

Wood and wood-derived materials have recently attracted attention as sustainable platforms for energy storage and conversion devices [125,126,127,128,129,130,131]. The intrinsic hierarchical porosity, high surface area, and rich chemical functionality of cellulose, lignin, and hemicellulose enable the fabrication of electrodes, separators, and conductive scaffolds with enhanced electrochemical performance. Carbonized wood structures, preserving the natural aligned channels of the cellular architecture, offer efficient pathways for ion and electron transport, which are critical for high-rate and high-capacity energy storage.

Natural wood veneers have been successfully employed as porous, lightweight substrates for supercapacitor composite electrodes, leveraging their hierarchical cellular structure, penetrating channels, and hydrophilic character. Lyu et al. [132] integrated polyaniline/reduced graphene oxide (PANI/RGO) and polypyrrole/reduced graphene oxide (PPy/RGO) onto wood substrates using physical deposition and in situ polymerization, yielding electrodes with high gravimetric specific capacitances of 931.92 F g^−1^ and 848.01 F g^−1^, respectively, at a current density of 2.5 mA cm^−2^ in three-electrode tests. Symmetric supercapacitors assembled from these electrodes exhibited areal specific capacitances of 0.89 F cm^−2^ and 0.78 F cm^−2^ at a sweep rate of 1 mV s^−1^, along with areal specific energies of 107.70 mW h cm^−2^ and 86.96 mW h cm^−2^ at an areal specific power of 0.25 mW cm^−2^. Both electrodes maintained excellent cycling stability, demonstrating the capability of wood-based substrates to preserve structural integrity while enabling high-performance electrochemical functionality.

**Figure 10:**
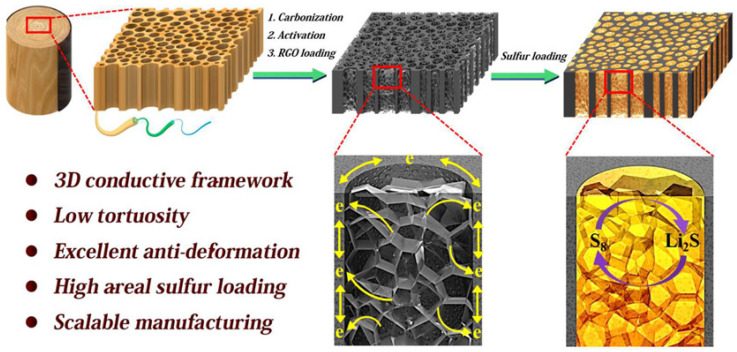
Schematic illustration of the preparation process and structure of the S@C-wood composite electrode. Reproduced with permission [133]. Copyright 2017. Reproduced with permission from American Chemical Society.

As illustrated in Figure 10, Hu et al. [133] developed a lithium–sulfur battery cathode inspired by the natural three-dimensional architecture of wood. The cathode comprises aligned microchannels filled with reduced graphene oxide, creating a 3D porous carbon matrix that serves as a lightweight, conductive, and structurally robust current collector. This architecture supports high sulfur mass loading, enabling the battery to reach an areal capacity of 15.2 mAh cm^−2^ with a sulfur content of 21.3 mg cm^−2^. Xie et al. [134] reported an all-wood-structured asymmetric supercapacitor (ASC) that leverages carbonized wood for high-performance energy storage. The device integrates an activated wood carbon anode, a thin wood membrane separator, and a MnO_2_/wood carbon cathode. The natural wood architecture provides multi-channel pathways, low tortuosity, high ionic and electronic conductivity, and structural stability, enabling exceptionally high areal mass loadings of up to 30 mg cm^−2^ for the anode and 75 mg cm^−2^ for the cathode. These structural advantages translate into a high areal capacitance of 3.6 F cm^−2^, energy density of 1.6 mW h cm^−2^, maximum power density of 24 W cm^−2^, and long cycling stability, representing the highest performance among MnO_2_-based supercapacitors. All components are low-cost, environmentally friendly, and biocompatible.

Yang et al. [27] developed flame-retardant wood-based composite phase change materials (PCMs) by coating delignified wood/PEG-1000 structures with PDMS/expanded graphite (EG), achieving simultaneous enhancements in flame retardancy and energy storage performance. The modified composites maintained high PEG-1000 loading mass fractions of 53.1–73.4%, ensuring substantial latent heat storage with phase change temperatures of 30–32 °C. Flame retardancy was significantly improved, with pHRR reduced by 40.1–52.3% and THR decreased by 12.3–16.1% compared with PEG-1000@DW. Importantly, when EG content exceeded 0.5 g, the composites self-extinguished during combustion and preserved structural integrity without PCM leakage. The composites also exhibited excellent cycle stability, retaining thermal reliability after 100 heating-cooling cycles. Moreover, owing to the strong light absorption and thermal conductivity imparted by EG, the materials demonstrated remarkable photothermal conversion efficiencies of 68.1–80.0% and enhanced radial thermal conductivity by 2.18–4.3 times relative to PEG-1000@DW. These results highlight that integrating EG-based coatings not only addresses the inherent flammability of wood-based PCMs but also unlocks their potential for solar energy storage and advanced thermal management applications.

## 7. Concluding Remarks and Future Directions

Over recent decades, wood has transformed from a conventional structural material into a versatile high-performance platform through advances in hierarchical structural engineering, chemical functionalization, and nanoscale modification. Techniques including delignification, densification, surface coatings, impregnation, and nanocellulose extraction have enabled precise control of the mechanical, thermal, and functional properties of wood. These strategies have addressed the persistent limitations of natural wood, including its flammability, dimensional instability, and susceptibility to biological degradation, while developing opportunities to introduce multifunctional capabilities such as flame retardancy, electromagnetic interference shielding, energy storage and photothermal conversion.

Flame-retardant functionalization has emerged as a particularly transformative development. Approaches that combine molecular-level and nanoscale modifications enhance fire safety, structural integrity, and environmental compatibility. The incorporation of phosphorus, nitrogen, and silicon-based additives, hybrid inorganic–organic coatings, and metal-ion-catalyzed char formation illustrates the effectiveness of synergistic strategies in producing wood with fire resistance comparable to that of synthetic composites while maintaining sustainability. Nanocellulose-based modifications and in situ polymerization within delignified scaffolds provide additional opportunities for multifunctional materials, combining lightweight architectures with tailored conductivity, thermal management, and mechanical performance.

Despite significant progress, substantial challenges remain for the broad adoption of engineered wood. Critical issues include the scalability of chemical and physical modification techniques, economic competitiveness compared with synthetic alternatives, long-term stability under fluctuating environmental conditions, and the complete environmental compatibility of additives. The complex interaction between multifunctional performance, structural anisotropy, and hierarchical architecture necessitates a more comprehensive mechanistic understanding and the development of predictive design strategies.

Mechanical performance also remains a critical factor, particularly for applications in construction and structural components. Strength, stiffness, toughness, and long-term dimensional stability must be optimized alongside functional enhancements to ensure reliability under real-world loading and environmental conditions. Future research should therefore focus on strategies that simultaneously enhance mechanical robustness and multifunctional performance, including densification, nanocellulose reinforcement, and hierarchical design principles.

Future research should focus on maximizing resource efficiency while enhancing mechanical and multifunctional properties. Strategies include hierarchical design principles, reinforcement with nanocellulose or other bio-based additives, and the development of fully bio-based flame-retardant and adaptive systems. In addition, increasing demand and unsustainable harvesting threaten biodiversity and impair the ecological functions of forests, including carbon storage, water regulation, and habitat provision. Efficient utilization of wood is essential to reconcile societal development with environmental protection, including the use of processing by-products and repurposing damaged or decayed trees. Advances in wood modification, recycling, and composite design will play a crucial role in enhancing material performance while minimizing ecological impact, supporting a sustainable and resilient bioeconomy.

At the same time, the comprehensive utilization and high-value conversion of bio-based raw materials to develop fully bio-based, formaldehyde-free flame-retardant and adhesive systems will be crucial for promoting sustainable development in the wood industry. In addition, there is an urgent need for the in-depth analysis and precise design of synergistic flame-retardant mechanisms. Although synergistic effects are widely discussed, their action mechanisms in multicomponent, multi-interface systems remain poorly understood, with many studies still limited to macroscopic performance characterization and speculative mechanism analyses. Future research should employ advanced in situ characterization techniques and multiscale computational simulations, such as synchrotron radiation, high-temperature Raman spectroscopy, and molecular dynamics, to monitor, in real time, radical quenching, char layer evolution, and mass and energy transfer across interfaces during combustion. While enhancing flame retardancy often reduces mechanical properties, increases costs, or introduces additional environmental concerns, future efforts should aim to resolve these performance trade-offs rather than simply stacking functions. For example, molecular structures could be designed to act simultaneously as flame retardants, plasticizers, or crosslinking agents, thereby compensating for mechanical losses.

Finally, investigating safety and intelligent functionalities throughout the entire life cycle represents an important frontier. Current research largely focuses on combustion performance, while long-term issues such as flame-retardant migration or leaching, performance degradation under photo or thermal aging, and the ecotoxicity of combustion byproducts have received insufficient attention. Consequently, establishing a comprehensive Life Cycle Assessment (LCA) framework is essential. In addition, integrating intelligent features such as sensing, responsive behavior, and self-healing with flame retardancy marks a significant step toward advanced wood-based materials. For example, developing smart flame-retardant wood composites with fire-warning capabilities, thermochromic damage indicators, or self-healing properties could open up considerable opportunities in high-tech applications, including smart homes, safety protection systems, and aerospace.

## Figures and Tables

**Table 1:** Comparison of material information and flame-retardant properties of wood modified with different treatments.

Composition of Materials	Preparation Method	Fillers Content	LOI (%)	PHRR (KW/m^2^)	THR (MJ/m^2^)	UL-94 Rating	Building-Code Taxonomy	Ref.
BA/ADP-FA-wood(FA/BA/ADP)	Impregnation	15 wt% FA + 3.2 wt% BA + 0.8 wt% ADP	34.4	156.57 (−35.8%)	54.7 (−37.4%)	N/A	LOI: GB/T 2406.2-2009	[77]
WS-SsSt11(Ss/St)	Impregnation	5%Ss + 5%St	N/A	190.1 (−31.0%)	57.2 (−14.9%)	N/A	Cone calorimeter: ISO 5660:2015	[78]
DW-GP/BA(GP/BA)	Impregnation	GP:BA = 10:1	N/A	9.2 (−89.5%)	1.4 (−78.46%)	N/A	Cone calorimeter: ISO 5660-1:2016	[79]
MW_22_(ZnB)	In situ mineralization	22.1 wt% ZnB	41.2	58 (−46.9%)	36.5 (−47.9%)	N/A	LOI: ASTM D2863-2017; Cone calorimeter: ISO 5660-1(2002)	[80]
S/C-wood(SA/CaCO_3_)	In situ mineralization	29 wt% CaCO_3_ + 1 wt% SA	N/A	173.42 (−59.51%)	24.39 (−48.52%)	N/A	N/A	[81]
AMP/FA-wood(FA/AMP)	Reactive grafting and covalent bonding	20 wt% FA + 8 wt% AMP	36.3	96 (−72.2%)	36.3 (−48.9%)	V-0	LOI: ASTM D2863-97; Cone calorimeter: ASTM E1354-2017; UL94: ATSM D3801-10	[82]

Note: The abbreviations used in the table are defined as follows: N/A indicates data that is not available. Abbreviations: BA: boric acid; ADP: ammonium dihydrogen phosphate; FA: furfuryl alcohol; Ss: sodium silicates; St: sodium tetraborate; WS: wood-scrimber; DW: delignified wood; GP: guanidine phosphate; ZnB: zinc boride; SA: sodium alginate; MW: mineralized wood; AMP: ammonium phytate.

## Data Availability

No new data were created or analyzed in this study. Data sharing is not applicable to this article.
